# Editorial: Spirituality and religion: implications for mental health

**DOI:** 10.3389/fpsyg.2025.1690862

**Published:** 2025-11-26

**Authors:** Angie Cucchi, M. Walid Qoronfleh

**Affiliations:** 1 School of Social Sciences and Professions, London Metropolitan University, London, United Kingdom; 2 Healthcare Research & Policy Division, Q3 Research Institute (QRI), Ann Arbor, MI, United States

**Keywords:** spirituality, religion, mental health, wellbeing, neurotheology, resilience, culture, psychotherapy

## Introduction

Spirituality and religion (S/R) have been central to human experience for centuries, providing frameworks for understanding life's purpose, coping with adversity, and fostering connections with oneself, others and the divine. Yet, it is only in recent years that empirical research has begun to systematically pay attention to these domains and recognize them as integral to understanding the full spectrum of human psychological experience. Furthermore, while in the past the integration of these constructs into psychological research primarily focused on philosophical debates, recent scholarship has recognized the relevance of these dimensions in shaping mental health outcomes worldwide (Mueller et al., 2001; Koenig, 2009; NAMI, 2016; PT, 2010). As such, increased scholarly attention has been given to the intersection between spirituality, religion, and mental health, acknowledging both the potential benefits, and challenges.

Many people's core values are shaped by S/R; for many individuals, S/R offer a sense of belonging, hope, and meaning that can act as a buffer against mental health difficulties. This protective factor has been shown to positively impact depression rates, the incidence of suicide attempts and generally improve wellbeing (Aggarwal et al., 2023; Lucchetti et al., 2021). S/R beliefs can be a source of profound comfort for some individuals. At the same time, research also suggests that rigid religious or spiritual beliefs can trigger feelings of intense inner and interpersonal conflict, especially when one's inner lived experience clashes with these teachings. This dissonance has been shown to exacerbate some mental health difficulties and to adversely shape the phenomenology of mental health disorders (Lucchetti et al., 2021). In addition, an over-reliance on S/R has been shown to negatively impact help-seeking behaviors, delaying or even discouraging engagement with mental health services (Akther et al., 2025).

Far from reaching a universal agreement on the intersection between S/R and mental health, the current body of evidence highlights the utmost need to continue to provide spaces where such conversations can be held. This is especially important because the complex mechanisms that lead some individuals to adopt S/R as either a negative, or a positive coping mechanism have not yet been fully understood. Recognizing these processes would enable clinicians to develop targeted treatment pathways to support individuals who wish to engage with and hold S/R in positive regard. Interestingly, recent research has emerged about specific biological markers associated with positive S/R (Lucchetti et al., 2021). Although in its infancy, these findings present an interesting avenue to further explore the relationship between S/R and mental health.

Building on this emerging evidence is the field of neurotheology, the scientific study of the neural processes that underlie religious and spiritual experiences and how these shape cognitions, emotions, and behavior (Newberg, 2025). A systematic review submitted to our Research Topic presented the convergence of neuroscience and S/R in the context of mental health (Haverkamp et al.). From a neurobiological standpoint, this relationship has been investigated using genetics, neuroanatomical, neurochemical, and functional techniques, in particular, neuroimaging technologies like MRI, fMRI, & PET. Indeed, functional neuroimaging has been an instrumental tool as it identified several brain areas whose activity is correlated with S/R, but whether these regions are causally involved in these behaviors is unknown. Despite some scientific limitations, the research findings suggest that different spiritual practices activate several brain regions.

A comprehensive analysis (Rim et al., 2019) of the literature identified the activation of the medial frontal, the orbitofrontal, the precuneus and the posterior cingulate cortexes, as well as the default mode network, and the caudate nucleus during various religious practices such as mystical experiences, prayers (either high- or low-structured), the recitation of religious texts, and the evaluation of religious statements and moral dilemmas. These findings suggest that spiritual/religious experiences and thinking about religious matters activate areas that are implicated in the processing of emotion, self-representation, reward pathways, and cognitive processing, suggesting an important role of S/R in these aspects of an individual's life. Building on this evidence, McNamara and Grafman (2024) propose an integrative framework, the Triple Network or Tripartite Network Model (TPM), that argue that spiritual/religious experiences depend upon the interactions of these regions, specifically the default mode network (DMN), the frontoparietal network (FPN), and the salience network (SN).

Extending this evidence to clinical settings, Rosmarin et al. (2022) explored neurobiological correlates in the context of depression, anxiety, substance misuse, and psychosis. In cases of clinical depression, analysis revealed that S/R was associated with greater cortical thickness and/or higher pial surfaces, in addition to increased white matter. Greater cortical thickness and pial surfaces are associated with higher executive functions such as decision making, abstract thinking and a more sophisticated brain function while the latter is linked to enhanced connectivity between brain regions. Interestingly, contrasting findings were found in a few substance misuse studies, where frequent Ayahuasca users were reported to display lower cortical thickness in correlation to higher self-transcendence and S/R. While it can be argued that Ayahuasca facilitates spiritual/religious experiences, it also seems plausible that it precipitates structural brain changes that differ from those found in S/R experiences without Ayahuasca use.

The same review also found that individuals who prayed regularly experienced less alcohol cravings and demonstrated increased activation of middle & left-brain regions implicated in executive functions, decision-making and emotional regulation. However, the evidence is still limited and still largely inconsistent. For example, in psychosis higher intrinsic religiosity was associated with decreased left medial, right medial, and right lateral orbitofrontal cortex when compared to controls. Furthermore, “lesion network mapping”- a relatively new neuroimaging technique- found that lesion sites associated with S/R beliefs map onto a specific brain circuit: the periaqueductal gray (PAG), a brainstem region implicated in fear conditioning, pain modulation, and altruistic behavior (Ferguson et al., 2022). This suggests that S/R activates areas important for managing fear and pain, as well as areas that foster prosocial behavior and attachment bonds. Consistent with these findings, S/R has been observed to increase under adverse circumstances, providing meaning, a buffer against pain, and emotional/practical support. Taken together, these aspects can contribute to better mental health and wellbeing.

Our Research Topic of *Frontiers in Psychology* positioned itself at the convergence of these dialogues and aimed to create a platform for different perspectives and interdisciplinary contributions to be shared. The ultimate goal was to start challenging the historical divide between modern psychology and spiritual/religious ideologies by encouraging conversations grounded in evidence-base and to explore more holistic models of wellbeing.

While this editorial does not aim to comprehensively summarize the myriads of contributions to the Research Topic, a few themes can be highlighted. These themes support the bulk of evidence discussed above. For example, a reoccurring message is that the experience of divine love and a secure attachment to a divine figure can foster emotional regulation, self-forgiveness, and interpersonal connectedness, reducing loneliness in the general population and mediating the relationship between subjective wellbeing and life stressors in immigrants who experience cultural displacement. In line with that discussed overleaf, these findings were also corroborated by the evidence that praying can lead to neurocognitive changes associated with more effective emotional regulation and better interpersonal connectedness. Additionally, several studies emphasized the importance of religious and cultural sensitivity, reporting the benefits of integrating culturally-congruent spiritual practices both with adults and children alike. These benefits seemed particularly evident during distressing times, such as the recent COVID pandemic or at times of serious illness.

Another prominent theme was the reliance on S/R for some individuals when a calamity befalls them, as well as how healthcare workers and caregivers manage health crisis. The findings of these studies support the effectiveness of spiritually based interventions in fostering patient's and healthcare workers' coping skills, resilience, and quality of life. These benefits are often the result of the role of S/R role in meaning-making, reduction of existential anxiety and instillation of hope, factors that have long been documented as crucial for resilience and post-traumatic growth (Frankl, 1962). In our editorial, this effect has been shown to be particularly relevant in cases of communicable diseases like COVID-19 (3-papers), as well as non-communicable diseases (NCDs- 10-papers) such as cancer (Ahmadi et al.), neurological disorders (Mameniškiene et al.), or cardiac disease (Amin). The relevance of S/R extends beyond illness contexts, with several papers also highlighting the importance of S/R for the elderly (Stelcer et al.), as well as in instances of workplace violence or bullying (Vveinhardt and Deikus).

In addition to these findings, an additional dimension that emerged was the ways in which cultural diversity and different philosophical frameworks informed mental health beliefs and practices, influencing the choice of adaptive coping strategies and defense mechanisms that individuals develop and use. For instance, in the Middle East there is a long-standing Islamic spiritual tradition of practices that are now described as cognitive challenging/restructuring and mindfulness (Al Balkhi, as cited in Badri, 2013; Al Ghazali, as cited in Waley, 2021, Ghuloum et al.). Additionally, religious ideologies and cultural beliefs shape perceptions of mental health and traditional and faith healers remain very popular and are often the first line of treatment for people experiencing mental health difficulties. Comparison studies between the Indonesian (Tanaka et al.), Japanese, Black Caribbean (Skalski-Bednarz et al.), Vietnamese Confucian (Giang et al.), Chinese Buddhist culture (Tan et al.), and indigenous shamanic communities (Sun and Kim) confirm that the processes that range from the recognition of mental illness and causal attribution to the selection of coping strategies can vary considerably across cultures, religions, and countries.

The submitted research articles highlight that religion and spirituality should not be seen as a peripheral matter, as they are often embedded in some individuals' and communities' psychological make-up and daily lived experiences. The contributions to the Research Topic emphasize the importance of adopting a religious and/or spiritually- inclusive lens when addressing mental health concerns, when appropriate. This view mirrors an increased recognition by both mental health professionals, as well as scholars, of the importance of understanding patients' spiritual and religious beliefs to develop personalized treatment plans that respect individuals' values system, enhance engagement, and contribute to the decolonization of the mental health field. Indeed, over the past few decades, psychology has expressed and shown a commitment to tackling systemic inequalities and dismantling colonial structures and practices (APA, 2021).

Recognizing that many non-Western cultures have not undergone the same process of individual, social and institutional secularization as the West means embracing spirituality as an integral dimension of human experience within these cultural contexts (Fernando, 2019). It means recognizing that often, in the African, Asian and Pacific Rim regions, the medical approach does not exclude the religious, or philosophical one: the human experience is conceptualized in more holistic terms that go beyond the bio-psychosocial model. Appreciating these cultural nuances fosters a more ethical and moral practice, one where cultural biases and hegemony are challenged while humility and inclusivity are embraced. This is indeed in line with the APA's mission statement (APA, 2021).

Although not a deliberate editorial choice, it is noteworthy that virtually all the studies submitted to this Research Topic emphasized the positive elements of S/R engagement. Although the protective elements of S/R are an undeniable finding, the editors need to consider whether the call for papers may have inadvertently embodied an inherent bias that attracted more submissions from this perspective. If this is the case, the unintended consequence would be the exclusion of more critical, ambivalent, and possibly detrimental dimensions of S/R that are, nevertheless, well-documented in the existing literature (Lucchetti et al., 2021). This could be an important limitation of this Research Topic, highlighting the need to maintain openness in future calls for research and ensure a comprehensive exploration of the multi-dimensional aspect of S/R engagement, including both the potential benefits and psychological risks.

The few studies that indirectly featured negative spiritual or religious coping (NSPC) as part of a broader investigation reported a significant negative impact of internal spiritual struggles on health-related quality of life (HRQoL) in cancer patients (Onyedide et al.), and in older adults (Stelcer et al.). The impact was reported on multiple aspects of wellbeing, including emotional, physical, social, and functional. Interestingly, the social/family dimension was reported to be particularly vulnerable to the effects of negative S/R coping, suggesting important relational consequences of S/R struggles, perhaps due to a disruption in the support systems. Furthermore, NSPC was shown to entirely mediate the relationship between maladaptive cognitive patterns and HRQoL, with age also impacting this association. Older adults were found to be more vulnerable to physical and functional poor health when experiencing spiritual distress.

The impact of spiritual struggles on older adults' mental health was also supported by one of the studies (Stelcer et al.) in this Research Topic. Given that older adults are already particularly vulnerable to social isolation and a disruption of interpersonal relationships (Ran et al., 2024), it seems crucial to explore what mechanisms might underline S/R struggles in this population to foster a more adaptive S/R attachment/engagement, when appropriate. Interestingly, even positive practices such as forgiveness, or a deeper personal religious history were associated with an increase in internalized self-blame and a decrease in an individual's ability to put things into perspective, which in turn has been shown to negatively impact (Brečka et al.) mental health. Far from being random occurrences, S/R struggles have been shown to be mediated by attachment insecurity (Freund et al.).

There is preliminary evidence to suggest that an individual's attachment to God may mirror the person's overall attachment system, leading them to feel that as others may have been unavailable or distant, or punishing, God may be the same. Interestingly, attachment insecurity did not predict S/R wellbeing, suggesting that S/R positive and negative coping strategies may arise from different psychological mechanisms (Freund et al.). The above findings hint at the nuanced, complex, and multifaceted impact of S/R on mental health and coping, demonstrating that some aspects of S/R can positively impact on wellbeing whereas others may exacerbate mental health difficulties. The findings highlight the need for researchers and clinicians alike to move beyond simplistic assumptions of S/R. Attention should be given to individual differences and the ways in which spiritual beliefs interact with attachment patterns, coping styles, and other bio-psychosocial factors. Future research should also concentrate on exploring how S/R frameworks might be tailored to support psychological wellbeing while mitigating potential risks. This would support mental health practitioners to adopt a person-centered approach to better integrate S/R into assessment and intervention, ultimately fostering equity and social justice.

## Conclusion

It is estimated that over 80% of the global population consider themselves spiritual or religious. Spirituality and religion have significant implications for mental health, with research suggesting both positive and negative effects depending on individual and cultural factors. Higher S/R has been associated with better outcomes for a variety of psychological presentations, though negative religious coping can worsen symptoms. Recent developments offer interesting insights, particularly in the field of neurotheology and neuroscience, although these warrant further attention. As research continues, mental health professionals must remain sensitive to the diverse ways in which S/R might shape mental wellbeing. Clinicians should consider S/R in treatment plans to provide holistic care and to ensure a decolonizing stance, one that regards all knowledge as equally valuable. By incorporating spiritual and religious perspectives, when appropriate, clinicians and researchers alike will contribute toward a more holistic, equitable, and socially just approach to mental wellbeing.
